# Injury severity at the time of sport-related ankle sprain is associated with symptoms and quality of life in young adults after 3–15 years

**DOI:** 10.1080/07853890.2023.2292777

**Published:** 2023-12-13

**Authors:** Oluwatoyosi B. A. Owoeye, Juan Paz, Carolyn A. Emery

**Affiliations:** a Department of Physical Therapy and Athletic Training, Doisy College of Health Sciences, Saint Louis University, St Louis, MO, USA; b Sport Injury Prevention Research Centre, Faculty of Kinesiology, University of Calgary, Calgary, Alberta, Canada; c Center of Excellence in Maternal and Child Health Education, Science and Practice, College for Public Health and Social Justice, Saint Louis University, St Louis, MO, USA; d McCaig Institute for Bone and Joint Health, Cumming School of Medicine, University of Calgary, Calgary, Alberta, Canada; e Alberta Children’s Hospital Research Institute, University of Calgary, Calgary, Alberta, Canada; f Department of Community Health Sciences, Cumming School of Medicine, University of Calgary, Calgary, Alberta, Canada; g Department of Pediatrics, Cumming School of Medicine, University of Calgary, Calgary, Alberta, Canada; h Brien Institute for Public Health, University of Calgary, Calgary, Alberta, Canada

**Keywords:** Ankle injury, chronic ankle instability, injury severity

## Abstract

**Background:**

Ankle sprains are the most common sports-related injuries. Individuals with time-loss ankle sprains often experience residual symptoms and chronic ankle instability years after injury. Up to 90% of post-traumatic ankle osteoarthritis cases are associated with severe ankle sprain. This study aimed to examine whether ankle injury severity sustained during youth sports participation is associated with ankle symptoms and function.

**Materials and Methods:**

Cohort study included 50 young adults (mean age, 23 years) with a 3-to 15-year history of a youth-sport related ‘significant ankle sprain’ (SAS). The primary independent variable was injury severity, which was captured in the index SAS injury details through interviews. SAS was defined as ligament and other intra/extra-articular structure injuries that disrupted youth sport participation, at least 3 days of time loss, and required medical consultation. Severe SAS was defined as SAS involving >28 days of time loss, and non-severe SAS only involved ankle ligaments and/or with ≤28 days of time loss. The Foot and Ankle Outcome Score questionnaire was used to assess ankle symptoms and function. Descriptive statistics and multivariable linear regression models were used to examine the association between SAS severity and outcomes, with sex and time since injury as covariates.

**Results:**

Compared to participants with non-severe SAS, participants with a history of severe SAS demonstrated significantly poorer outcomes in symptoms [-18.4 (99% CI: −32.2 to −4.6)], pain [-10.1 (99% CI: −19.2 to −1.1)] and QoL [-17.1 (99% CI: −33.1 to −1.1)] in multivariable linear regression models.

**Conclusions:**

Severe ankle sprain with a loss of > 4 weeks from sports participation at the time of injury is independently associated with poorer ankle symptoms, pain, and ankle-related quality of life after 3–15 years. Secondary prevention measures are needed in individuals with a history of severe ankle sprains to mitigate the potential health consequences.

## Introduction

There are many health benefits associated with regular physical activity participation within the life span [1–3]. However, participation in physical activity is associated with the risk of musculoskeletal injuries [4]. Ankle sprains are the most common sport-related injury, with an annual incidence of 2.2–3.3 injuries per 1000 person-years when considering emergency department data from the National Electronic Injury Surveillance System [5–7]. Ankle sprain injury is the most common injury in youth sports and physically active individuals of all ages and most frequent in team sports with repeated jumping/landing and change of direction [8–12]. Ankle sprain injury accounts for an incidence of 13.6 injuries per 1000 athlete exposures in females and 6.94 per 1000 athlete exposures in males [10].

Following a lateral ankle sprain, 30–75% of individuals reportedly experience residual symptoms and perceived instability ranging from 1to 4 years after the initial injury [13–15]. These ankle-related symptoms may persist up to an average of 8.6 years among individuals [16]. Up to 90% of posttraumatic ankle OA is associated with severe ankle sprains [17]. Injury severity at the time of injury may be an important indicator in the etiology of posttraumatic ankle OA years after injury. Foot and Ankle Outcome Score (FAOS) is a popular and valid patient-reported outcome measure used to assess symptoms and function in individuals with osteoarthritis and other foot- and ankle conditions [18–20]. It is regarded as the most responsive tool in patients with ankle instability and ankle OA [19,20].

To reduce the burden of chronic ankle instability and persisting ankle problems after an ankle sprain injury, and to mitigate the potential risk of post-traumatic osteoarthritis OA of the ankle, a potential long-term problem, there is a need for more research [21]. Understanding the dynamics in the risk profiles of individuals with previous time-loss ankle sprain injury will guide secondary prevention measures that will ultimately reduce the burden of ankle sprains and associated consequences. The objective of this study was to determine whether ankle injury severity is associated with ankle symptoms and function in young adults with a history of ankle sprains. We hypothesized that a more severe injury will result in increased ankle symptoms and reduced function over time.

## Materials and methods

### Study design

This study was a sub-cohort secondary analysis of a larger cohort study [16] that evaluated the association between youth sport-related ankle sprain injury and health-related outcomes, 3–15 years post-injury. Fifty male and female young adults with a history of ankle sprain from soccer and/or basketball participated in this study. All participants provided written informed consent before participation, and the study was approved by the Conjoint Health Research Ethics Board (REB16-2280) of the University of Calgary, Canada.

### Data collection

Eligible study participants were prescreened *via* extensive phone interviews by a trained research assistant before inclusion in the study. Participants’ inclusion was based on meeting the study criteria of a history of a ‘significant ankle sprain’ (SAS) during participation in youth sport (in this case, soccer and/or basketball) 3–15 years prior and not having any other significant lower extremity injuries. Specifically, we defined SAS as a clinical diagnosis of an ankle ligament injury involving the lateral ligament, ligaments of the tibiofibular syndesmosis (high ankle sprain), and/or medial ligament sprain that resulted in disruption of regular youth (i.e. ≤18 years of age) sport participation, missing at least 3 days of time loss from sport or exercise participation, and required medical consultation (physician, physical therapist, and/or athletic trainer), 3–15 years prior to enrolment in this study [16,22]. As part of the interview, participants were asked to provide details of an ‘index’ SAS (i.e. their first SAS in the case of multiple), including approximate date/year of injury, diagnosis, medical attention received, time-loss duration, and information on additional/recurrent SAS injuries. Information regarding any significant injuries to the other parts of the lower extremities was also collected. Individuals reporting any recent significant lower limb injuries (as defined for SAS above but in relation to the entire lower limb) within the past 3 years and any history (past 12 months) or ongoing chronic conditions that may affect mobility and physical function were excluded.

The primary independent variable in this study, injury severity, was captured in the index SAS injury details. Injury severity was described as either ‘severe’ or ‘non-severe’, and this was based on a combination of SAS diagnosis at the time of injury (either from available medical record or self-report) and/or the time-loss duration provided by the participant [16]. We defined a severe SAS as ones affecting ankle ligaments and other intra/extra articular structures (e.g. bones and tendons) and/or with > four weeks of time-loss and non-severe SAS as injuries only affecting ankle ligaments and/or with ≤ four weeks of time-loss.

On enrolment, participants completed a preparticipation questionnaire and the FAOS questionnaire, and completed the baseline testing procedure, including body weight and height measurements, as part of a larger study [16]. The preparticipation questionnaire comprised demographics, musculoskeletal injury history (all injuries), and medical and sports/recreational activity participation history (within the past 12 months). The study outcomes, ankle symptoms, and functions were captured in the FAOS questionnaire. The FAOS was used to evaluate ankle-related symptoms and function among the participants (i.e. self-report, with reference to the week before testing). The FAOS is a tool that has been demonstrated to be valid and reliable in assessing foot- and ankle-related outcomes [18–20,23–30]. The FAOS consists of 42 items, with scores between 0 and 4, divided into 5 subscales: pain (9 items), other symptoms (7 items), activities of daily living (17 items), quality of life related to the ankle and foot (4 items), and level of participation in sports and recreation (5 items) [23].

### Statistical analysis

Statistical analyses were performed using Stata (version 16.1, College Station, TX, USA). Participant characteristics were described using mean ± standard deviation, median (range), frequency, and proportions, as appropriate. The association between injury severity and study outcomes (FAOS sub-categories) was assessed using multiple linear regression models considering the time since index SAS injury and sex (variables that may influence the relationship between injury severity and FAOS outcomes) as covariates. To adjust for a potential Type 1 error due to multiple hypothesis testing in the five FAOS subcategories, we used an alpha level of 0.01 (Bonferroni correction, 0.05/5) and the corresponding 99% confidence interval (CI) in the multivariable linear regression models.

## Results

All 50 participants (mean age: 22.9 ± 3.6 years; 80% female) completed the questionnaires. The participant characteristics are presented in Table 1. Forty-two percent (38% among females and 60% among males) of the participants had at least one severe SAS injury.

**Table 1.** Participant characteristics.

Variable	Female *n* = 40	Male *n* = 10
Age at follow-up (years)	23 (17–30)	23 (18–30)
Height (cm)	165 (146–183)	180 (158–193)
Weight (kg)	62 (47–83)	78 (69 –108)
Body Mass Index (kg/m2)	23 (19–30)	24 (21–34)
12-month history of sport/exercise participation n (%)	38 (95)	9 (90)
History of other ‘non-significant’ LLI, n (%)	6 (15)	2 (20)
Participants with multiple SAS (>1), n (%)	24 (65)	5 (56)
Index SAS severity – severe, n (%)	15 (38)	6 (60)
Time since index SAS, years	8.9 (3.1–15.0)	7.6 (3.6–14.5)

Values are median (range) unless noted.

SAS, significant ankle sprain: one that resulted in time loss and required medical attention.

Index SAS: the first SAS indicated by injured participants.

Non-significant: not resulting in time-loss from sport or exercise.

LLI, lower limb injury.

A significant association was found between SAS injury severity and three of the five FAOS clinical outcome measures. Compared with participants with non-severe SAS, participants with a history of severe SAS demonstrated significantly poorer outcomes in symptoms [-18.4 (99% CI: −32.2 to −4.6)], pain [-10.1 (99% CI: −19.2 to −1.1) and QoL [-17.1 (99% CI: −33.1 to −1.1)] (Table 2). The associations between SAS injury severity and all three clinical outcomes were independent of the time since the index SAS injury and sex. In the multivariable regression models, time since injury was not associated with any of the five clinical outcomes (*p* > 0.01), but sex was significantly associated with the FAOS subcategory outcome of symptoms, with male participants reporting higher scores than female participants (17.8 [99% CI:1.0–34.7); *p* = 0.007).

**Table 2.** Multivariable linear regression models showing associations between previous ankle injury severity and ankle/foot-related outcomes.

Variable	Constant β (99% CI)	Injury Severity β (99% CI)	p-Value
FAOS Symptoms	77.1 (68.3–86.0)	−18.4 (−32.2 to −4.6)	0.001*
FAOS Pain	90.3 (84.5–96.1)	−10.1 (−19.2 to −1.1)	0.004*
FAOS ADL	96.5 (93.4–99.3)	−0.5 (−4.9 to 3.9)	0.765
FAOS QoL	77.1 (66.8–83.3)	−17.1 (−33.1 to −1.1)	0.006*
FAOS Sports/Recreation	85.9 (78.0–93.9)	−11.2 (−23.7 to 1.2)	0.019

Final models were controlled for team clusters and adjusted for time since index SAS injury and sex (both covariates were not statistically significant in each of the five models).

Negative values indicate poorer outcomes in the case of FAOS.

β, beta coefficient: the mean difference between the categories of injury severity (severe minus non-severe).

Injury severity: severe (>4 weeks of time loss) vs. non-severe (≤4 weeks of time loss).

CI, confidence interval.

* Statistical significance at alpha = 0.01 (adjusted for five FAOS outcomes).

## Discussion

In this study, our goal was to determine the mid-term consequences of SAS injury severity, including symptoms and functional limitations, in individuals who experienced at least one SAS injury while participating in youth sports. Out of the five subscales of the FAOS, SAS injury severity was associated with three of the categories (pain, symptoms, and QoL), independent of time since index SAS injury or sex. Sex showed a significant relationship with the symptoms section of the FAOS, with men having poorer outcomes; however, time since injury had no relationship with any of the subscales.

Our findings regarding the association between previous ankle sprain severity and ankle-related outcomes are consistent with those of Thompson et al. [31] and Pacheco et al. [32] regarding prognostic factors associated with poor clinical outcomes in individuals with ankle injuries. Both studies listed injury severity as a clinical indicator for poor prognosis, but also determined that there is insufficient evidence at this point to offer any recommendations about clinical traits that would prognosticate a poor recovery or progressing disability. Further research is needed to accurately understand the prognosis and potential consequences of ankle sprains [32,33].

While it is well documented that ankle sprains can develop mid- and long-term dysfunction that persists for multiple years [13–17,34], not all ankle sprains end up as a disabling musculoskeletal condition related to post-traumatic ankle osteoarthritis, a potential primary consequence of an ankle sprain injury. Our findings contribute valuable knowledge for identifying individuals at high risk of future disabling joint conditions. Given the current evidence from imaging studies (ultrasound and MRI) demonstrating associations between clinical symptomatology and structural damage after suffering an ankle injury [33,35,36], it is possible that the poorer ankle-related outcomes exhibited in individuals with previous severe ankle sprain in our study are a result of progressive debilitating changes in both intra- and extra-articular ankle structures. It is also probable that some of these individuals already had structural damage to their ankle joints.

The sex difference in symptoms could be explained by the participant characteristics; 60% of the males reported suffering a severe sprain in comparison to females (38%). Furthermore, older female patients generally recover from injuries at a slower rate or incompletely compared to males [37]. Additional sex-related factors, including biopsychosocial factors, coping strategies, and goals/expectations, likely contributed to the sex difference observed [38].

This study had some limitations. Participants were interviewed regarding their history of ankle sprains and musculoskeletal lower-limb injuries over several years. This method of data collection is subject to recall bias and potentially affects the accuracy of our findings. To reduce the impact of this limitation, we repeated a few of the interview questions in our baseline study questionnaire and cross-checked the details of the interview information. Another limitation is that the FAOS instrument is not specific to the ankle, because each item asks about the foot and/or ankle. Nonetheless, the FAOS is endorsed by the International Ankle Consortium as one of the few responsive patient-reported outcome measures among the other measures currently available for ankle-related injuries [18,19,23–30] and it is currently recognized as the most effective tool for evaluating conservative treatment in patients with ankle OA [19,20]. Although multivariable regression analyses were used to find independent associations between ankle sprain severity and outcomes, we were limited by the total number of covariates we could add to the regression models given our sample size. There was a likelihood of residual confounding from other factors that were not considered. One such factor was the initial treatment received by the participants and the quality of rehabilitation. For example, a supervised home exercise program during rehabilitation demonstrated better outcomes than an unsupervised home exercise program or no home programs [39–41]. We did not collect information regarding details of rehabilitation in participants. Other potential confounders in our study include the number of ankle sprains in participants with multiple ankle sprains, types of footwear and history of other lower extremity injuries. Although we collected information on some of these variables, we did not have the statistical power to include all potential confounders in our multivariable regression analyses.

This study has several vital clinical implications. Based on our findings, individuals with ‘severe’ ankle sprains, defined in the current study as a time-loss injury of more than four weeks, may be classified as the ‘at risk’ population for future ankle-related chronic conditions with or without structural changes. Osteoarthritis is currently argued as not just a disease per structural joint changes, but also an illness with symptoms including pain and dysfunction that significantly impacts an individual’s overall health and quality of life [42]. Accordingly, any ‘pre-osteoarthritic’ outcomes demonstrated in individuals with severe SAS injury should be of concern. An evidence-informed framework for individualized periodic (e.g. every 1–2 years) ankle screening tests and evaluation to inform appropriate interventions will be a useful resource for clinicians managing musculoskeletal conditions. This may help relieve symptoms and improve quality of life among individuals and may also slow down the rate of progression into structurally diagnosed osteoarthritis.

## Conclusions

Independent of the time since injury, previous severe SAS injury, defined as an ankle sprain with a time loss of > 4 weeks, is associated with ankle symptoms, pain, and ankle-related quality of life 3–15 years following injury. Secondary prevention measures are needed in individuals with a history of severe ankle sprains to mitigate the potential health consequences.

## Data Availability

Available upon reasonable request.
